# Complete genome sequence of Koutango virus strain DakAnD5443 isolated from *Tatera kempi* in 1968

**DOI:** 10.1128/MRA.00423-23

**Published:** 2023-10-17

**Authors:** Gabriel J. Haila, Jessica A. Plante, Steven G. Widen, David W. C. Beasley

**Affiliations:** 1 Microbiology and Immunology Graduate Program, University of Texas Medical Branch, Galveston, Texas, USA; 2 Department of Microbiology and Immunology, University of Texas Medical Branch, Galveston, Texas, USA; 3 World Reference Center for Emerging Viruses and Arboviruses, University of Texas Medical Branch, Galveston, Texas, USA; 4 Department of Biochemistry and Molecular Biology, University of Texas Medical Branch, Galveston, Texas, USA; 5 Molecular Genomics Core Facility, University of Texas Medical Branch, Galveston, Texas, USA; 6 Institutional Office of Regulated Nonclinical Studies, University of Texas Medical Branch, Galveston, Texas, USA; 7 Sealy Institute for Vaccine Sciences, University of Texas Medical Branch, Galveston, Texas, USA; 8 Institute for Human Infections and Immunity, University of Texas Medical Branch, Galveston, Texas, USA; Katholieke Universiteit Leuven, Leuven, Belgium

**Keywords:** flavivirus, arbovirus

## Abstract

Koutango virus (KOUV), a close relative of West Nile virus, is highly neuroinvasive in animal models and has been associated with human disease. The complete genome of the KOUV prototype strain DakAnD5443 is reported here and may facilitate development of infectious clones for further characterization of this novel flavivirus.

## ANNOUNCEMENT

The prototype Koutango virus (KOUV) (*Orthoflavivirus koutangoense*) strain, DakAnD5443, was isolated from a wild rodent (*Tatera kempi*) in Senegal in 1968 (1). Phylogenetically, KOUV is a close relative and possible subtype of West Nile virus (WNV) (2) which was originally isolated in Uganda but which has emerged in the past 25 years as a significant cause of human and animal disease in the Americas and Europe. KOUV has only been detected in Africa, but studies in animal models show it has a highly virulent phenotype similar to that of WNV strains associated with epidemics of neurological disease (2 – 4). KOUV appears to differ from WNV in its arthropod vector preferences—including isolation from ticks and sandflies—and reservoir hosts have not been definitively identified (2, 5 – 9). Molecular virological studies of KOUV would be facilitated by development of infectious clones. However, only limited nucleotide sequence data are available for KOUV. Genomic sequences previously reported for KOUV strains cover only the virus open reading frame (ORF) or lack key terminal sequences in the untranslated regions (UTR).

KOUV DakAnD5443 obtained as a low passage stock (eight passages in suckling mice, one passage in Vero cells) from the World Reference Center for Emerging Viruses and Arboviruses was passaged once in Vero cells before RNA extraction using QIAamp viral RNA mini kit (Qiagen). Sequencing libraries prepared with the NEBNext Ultra II RNA Library Prep Kit were sequenced on an Illumina NextSeq 550 using the High-Output 75 base paired-end format. Reads were quality-filtered and adapter sequences were removed using Trimmomatic software (10), version 0.39 with parameters HEADCROP:5 ILLUMINACLIP:2:40:12 LEADING:35 TRAILING:35 SLIDINGWINDOW:5:35 MINLEN:35. Contigs were assembled using ABySS version 2.3.1 (11) with default paired-end parameters. Reads were mapped back to the contigs using bowtie2 (12) with the –local setting, and visualized with the Integrated Genomics Viewer (13) to verify that assembled contigs were correct. Sequencing obtained an average of 51,022 reads per base. To ensure complete end terminal sequencing, RNA ligase-mediated rapid amplification of cDNA ends (RLM-RACE) using the FirstChoice RLM-RACE Kit (Invitrogen) and 5´ template switching RACE using Template Switching RT Enzyme Mix (NEB) were performed, according to manufacturers’ protocols.

The complete genome of KOUV DakAnD5443 is 10,988nt (50.6% GC content) with a 96nt 5´ UTR and a 593nt 3´ UTR. Alignment of the complete KOUV genome with those of other Japanese encephalitis (JE) serogroup viruses using the Clustal Omega algorithm in MegAlign Pro, v.17.3.0, with default parameters indicates 77% nucleotide sequence similarity to representative lineage I (NY99, GenBank MZ605381.2) and II (B956, NC_001563.2) WNV strains, and 65%–71% similarity with representative JE (EF571853.1), Alfuy (AY898809.1), Usutu (AY453412.1), Saint Louis encephalitis (EU566860.1), and Murray Valley encephalitis (NC_000943.1) virus sequences. Nucleotide similarity to available ORF sequences of other KOUV strains ranged between 94.1% (PM148; MN057643.1) and 95.0% (ArD96655; KY703855.1).

**Fig 1:**
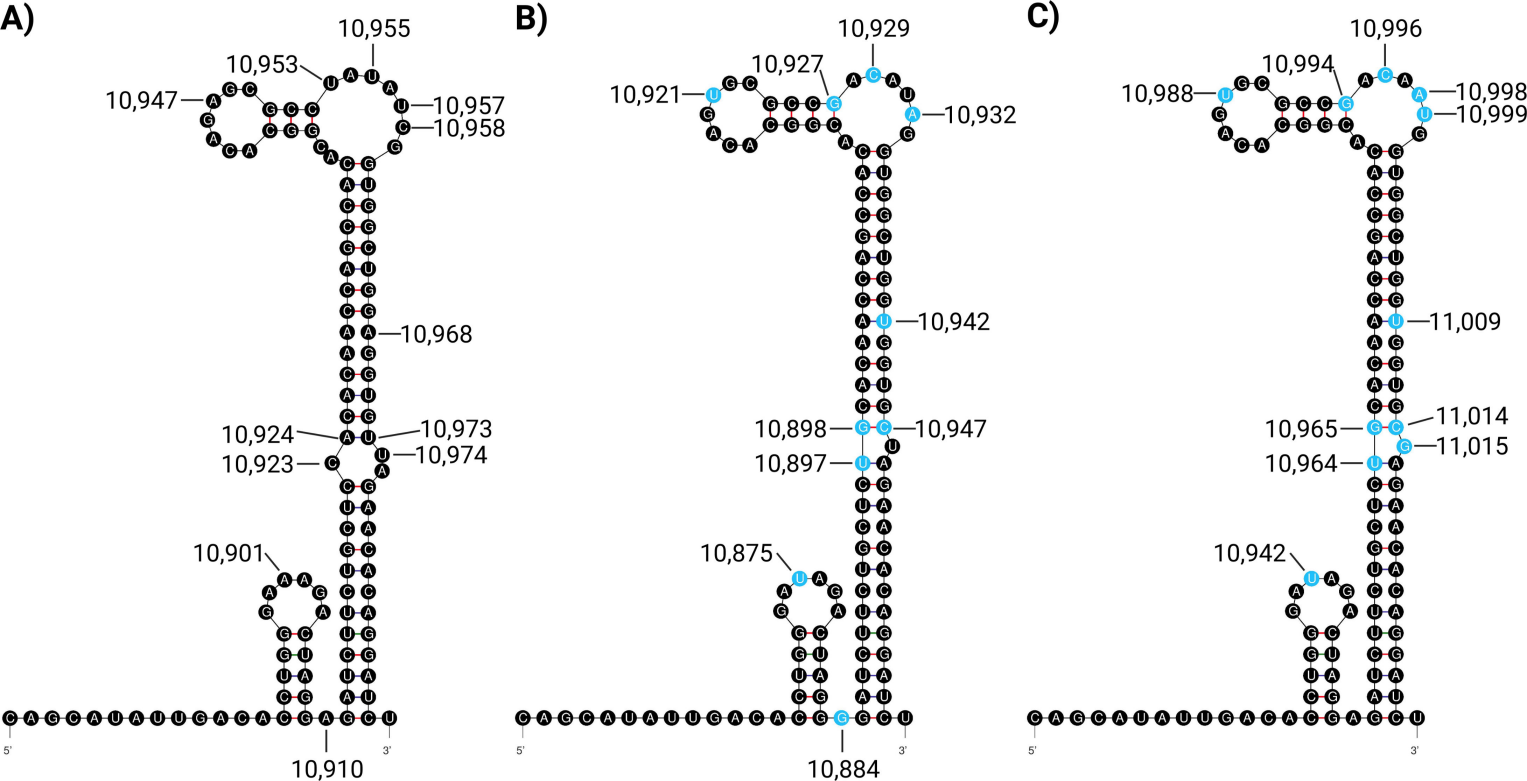
3´UTR terminal stem loop structures of (**A**) KOUV, (**B**) WNV B956, and (**C**) WNV NY99 predicted using mFold (16) and manually annotated using Biorender.com. Non-conserved nucleotides shown in blue in WNV structures.

Comparison of the 5´ and 3´ UTRs of KOUV to those of WNV NY99 and B956 revealed greater similarity to B956 but identified differences in highly conserved structures critical for virus replication. In particular, KOUV encodes mutations and altered secondary structures in the terminal 3´ stem loop in regions of the stem and top loop (Fig. 1) that were previously shown to affect replication efficiency of WNV 956 (14, 15), suggesting these sequences are not interchangeable between WNV and KOUV. Availability of the complete KOUV sequence will allow more detailed investigations of this understudied flavivirus.

## Data Availability

The complete KOUV DakAnD5443 genomic sequence has been deposited in GenBank under accession number OQ067500. Sequence data files have been deposited in the NCBI Sequence Read Archive under accession number PRJNA941375.
